# The Clinical Profile of Patients With Culture-Positive Urinary Tract Infections Admitting to a Tertiary Hospital in Sri Lanka

**DOI:** 10.7759/cureus.58666

**Published:** 2024-04-20

**Authors:** Sincy S Samarawickrama, Helika U Illangakoon, Ali Uthuman, Vinod Saranga, Chaminda Janaka

**Affiliations:** 1 Department of Medicine, Goulburn Valley Health, Shepparton, AUS; 2 Department of Medicine, University of Colombo, Colombo, LKA; 3 Department of Family Medicine, The National Hospital of Sri Lanka, Colombo, LKA; 4 Department of Rural Health, University of Melbourne, Shepparton, AUS; 5 Department of General Medicine, Goulburn Valley Health, Sehpparton, AUS; 6 Department of Surgery, Sri Jayewardenepura General Hospital, Sri Jayewardenepura Kotte, LKA; 7 Department of Medicine, Sri Jayawardenepuera Genral Hospital, Nugegoda, LKA

**Keywords:** antibiotics, klebsiella pneumoniae, escherichia coli, culture, urinary tract infection

## Abstract

Introduction

Urinary tract infections (UTIs) are globally prevalent. This study explores the clinical and pathological profile of culture-positive UTI patients at Sri Jayewardenepura General Hospital.

Method

In this descriptive cross-sectional study conducted at Sri Jayewardenepura General Hospital from December 2020 to May 2021, we evaluated patients over 14 years with positive urine culture reports. Excluding those with HIV, undergoing chemotherapy, or pregnant, we used consecutive sampling. Data were collected via interviewer-administered questionnaires and analyzed using SPSS version 21.0 (IBM Inc., Armonk, New York), employing descriptive statistics and Fisher's exact tests to identify factors associated with urinary tract infections.

Results

The study involved approximately 278 participants. The mean age remained 60 ± 20.279 years, with over half of the participants being female. Common symptoms like fever and lower abdominal pain were observed in 22.30% of cases. The incidence of acute kidney injury was 30.58%. *Escherichia coli* (36%) and *Klebsiella pneumoniae *(26%) were the predominant organisms found. Indwelling catheters and other urinary tract conditions were considered risk factors. Patients with at least one risk factor were more likely to receive antibiotics before the urine culture. Similarly, males exhibited a higher prevalence of at least a risk factor than females.

Conclusion

UTIs are a significant clinical issue in older populations, with females being more susceptible. Fever and abdominal pain were common symptoms. *Escherichia coli* and *Klebsiella pneumoniae* were the most frequent causative agents. Further research is necessary to identify risk factors and predictors of antimicrobial resistance in UTI patients.

## Introduction

According to recent statistics, urinary tract infections (UTIs) are among the most prevalent infectious diseases worldwide, affecting more than 150 million people annually [1]. It is linked to significant morbidity and health care costs across all ages, from newborn to elderly, and in both males and females. The etiological agents responsible for the illness are highly diverse as determined by factors like age, immune status, and underlying structural and functional abnormalities in the urinary tract [2]. However, in general, *Escherichia coli (E. coli) *is the most common pathogen responsible for the majority of urinary tract infections. Still, alarmingly extended-spectrum beta-lactamase (ESBL) resistance is an increasing problem among many populations, raising significant concerns during its management [3].

This study delves into the clinical and pathological profiles of culture-positive UTI patients admitted to a tertiary care center in Sri Lanka.

Urinary tract infections are diagnosed clinically supported by culture positivity with bacteriuria ≥10^5^ colony forming unit per milliliter (CFU/mL) in urine sampling with the clean catch method, more than 10^4^ in catheter sampling, and any colony count from suprapubic urine sampling in a symptomatic patient [4]. This will be coupled with signs of urinary tract inflammation as demonstrated by pyuria on urinalysis with >10 white blood cells (WBC)/mm^3^ per high-power field (HPF) [5].

The spectrum of UTI includes several clinical syndromes ranging from cystitis, pyelonephritis, and renal or perinephric abscess. Moreover, it can demonstrate a more complicated clinical picture leading to sepsis and death, particularly in immune-compromised people [6]. Consequently, the symptoms and complications of the disease are also highly heterogeneous depending on underlying risk factors and the individual's health status. Most of the time, the affected individuals may present with symptoms like burning micturition, urgency, dysuria, cramping in the lower abdomen, mental irritability, back or flank pain, chill, nausea, fever, vomiting, fatigue, and weakness [7]. According to the prevailing antecedents, these patients are broadly divided into two groups, complicated and uncomplicated, and the management principles depend on the type of UTI. Uncomplicated UTIs are described in individuals who are otherwise healthy and have no structural or neurological urinary tract abnormalities. They can be either upper UTI (pyelonephritis) or lower UTI (cystitis). On the other hand, complicated UTIs are linked to factors that compromise the urinary tract or host defense, including urinary obstruction, urinary retention caused by neurological disease, immunosuppression, renal failure, renal transplantation, pregnancy, and the presence of foreign bodies such as calculi, indwelling catheters or other drainage devices [8].

UTIs manifest in a broad range of symptoms, from mild discomfort to severe complications [3]. Commonly, patients report fever and dysuria [3], with additional symptoms like lower abdominal pain, groin pain, and headache being prevalent [9]. Urinary frequency and urgency are also noted [10]. Symptom onset to consultation typically spans three days, but longer durations and more severe symptoms are observed, especially in women with recurrent issues [11].

Blood and urine tests often reveal high inflammatory markers, such as erythrocyte sedimentation rate and C-reactive protein, indicative of upper UTIs [9]. *E. coli* remains the predominant pathogen in UTIs, with varied frequencies of other bacteria like *Klebsiella *and *Enterococcus *[3,12-14]. Antibiotic resistance is significant, with varying resistance rates to common antibiotics, presenting a pressing challenge [15]. In South India and Sri Lanka, high resistance rates to antibiotics like quinolones and penicillins have been reported, though some, like imipenem, retain effectiveness [3,16].

The diversity of UTI presentations and antibiotic sensitivities underscores the necessity for ongoing research to improve understanding and management [3,9-11,15-17]

## Materials and methods

Ethical approval and design

This study was approved by the Ethical Review Committee of Sri Jayewardenepura General Hospital (SJGH), ensuring adherence to ethical standards. Before commencement, we obtained permission from the hospital director and the ethical review board. A descriptive cross-sectional design assessed patients with positive urine culture reports at SJGH from December 2020 to May 2021. Documented informed consent was secured from all participants, affirming their voluntary participation and understanding of the study's purpose. Data were encrypted, access was password-protected, and all records were scheduled for deletion six months after the study's conclusion.

Inclusion criteria and data collection

Eligible participants were those admitted to SJGH, aged over 14 years, with positive urine culture reports, regardless of the presence or absence of urinary symptoms. Exclusions were applied to individuals diagnosed with HIV, those undergoing chemotherapy, and pregnant women with positive urine cultures to ensure the study's focus and reduce confounding variables. A sample size of 384 respondents was necessary for adequate power, based on a projected population proportion of 0.5 and an absolute precision of 0.05, using a consecutive sampling method for recruitment.

Data collection was facilitated through an interviewer-administered questionnaire meticulously conducted by the principal investigator to gather comprehensive patient data and ensure consistency.

Data processing and statistical analysis

The collected data were processed and analyzed using the Statistical Package for Social Sciences (SPSS) version 21.0 (IBM Inc., Armonk, New York). Through frequency distribution tables, descriptive statistics provided an overview of qualitative data, offering insights into the study population's characteristics. Fisher's exact test, among other statistical tests, was employed to examine the factors associated with urinary tract infections (UTIs) within a 95% confidence interval. This approach facilitated a detailed understanding of the patient profiles and the underlying patterns within the data.

## Results

**Figure 1:**
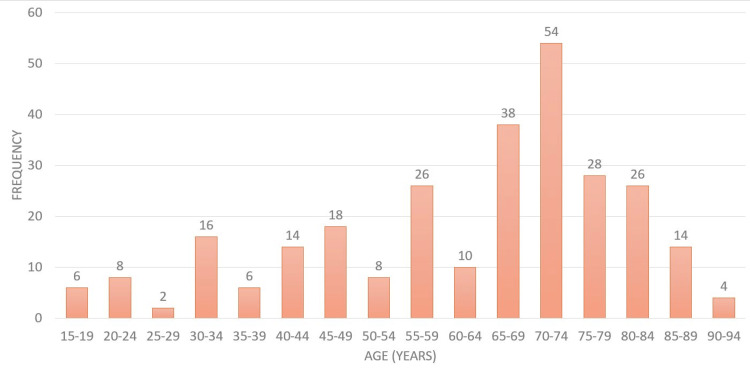
Histogram of the age distribution of the patient cohort

The study encompassed 278 participants, with their age distribution detailed in Figure 1.

The participants' ages ranged from 15 to 94 years, with the majority falling within the 70-74. The mean age of the study population was 60 ± 20.279 years. Regarding gender distribution, there was a predominance of female participants, accounting for 164 (59%) of the sample. Additionally, 236 (85%) respondents were married.

Table 1 shows the comorbidities among the study population. One hundred sixteen respondents, accounting for 41.72%, had hypertension, while 86 (30.93%) had diabetes. A smaller subset of 20 (7.19%) had experienced a stroke. Notably, a significant proportion of the sample (46.04%) reported no comorbid conditions.

**Table 1:** Comorbidities among the participants

Comorbidities	Number (%)
Diabetes	86 (30.93)
Hypertension	116 (41.72)
Ischaemic heart disease	42 (15.10)
Stroke	20 (7.19)
Chronic kidney disease	40 (14.3)
No comorbidities	128 (46.04)

Regarding the clinical presentation (Table 2), the most common symptoms reported were fever and lower abdominal pain experienced by 62 (22.30%) patients. Additionally, 34 (12.23%) patients had vomiting or nausea, while 32 (11.51%) patients reported dysuria and loin pain. Notably, a smaller number of four (1.43%) presented with perineal pain.

**Table 2:** Different clinical presentations in the study population

Clinical presentation	Number (%)
Fever	62 (22.30)
Dysuria	32 (11.51)
Urinary frequency	16 (5.75)
Urgency	8 (2.87)
Haematuria	6 (2.15)
Lower abdominal pain	62 (22.30)
Loin pain	32 (11.51)
Vomiting/nausea	34 (12.23)
Loss of appetite	24 (8.63)
Perineal pain	4 (1.43)

Regarding risk factors (Table 3), most respondents, 188 (67.63%), did not exhibit any. However, obstructive uropathy was noted as a significant risk factor, present in 30 (10.79%) patients, followed by renal calculi in 26 (9.35%) participants. A smaller subset of respondents, six (2.16%), had a history of uterovaginal prolapse or cystocele, and an even smaller number of two (0.72%) were identified with bladder dysfunction.

**Table 3:** Risk factors among the study group

Risk Factors	Number (%)
Indwelling catheter	22 (7.91)
Renal calculi	26 (9.35)
Obstructive uropathy	30 (10.79)
Renal cysts	4 (1.44)
Bladder dysfunction	2 (0.72)
Utero-vaginal prolapse/cystocele	6 (2.16)
None	188 (67.63)

The study's complications are summarized in Table 4. Acute kidney injury was the most frequent complication, affecting 85 (30.58%) patients. Altered levels of consciousness were noted in 28 (10.07%) patients, and septic shock was present in a smaller cohort of 7 (2.52%) patients. Notably, the majority, comprising 168 (60.43%) patients, experienced no complications.

**Table 4:** Complications as well as usage of antibiotics prior to the urine cultures among the study population

Variable	Number (%)
Complications	
Acute kidney injury	85 (30.58)
Altered level of consciousness	28 (10.07)
Septic shock	7 (2.52)
None	168 (60.43)
Antibiotics taken before urine cultures	
Yes	50 (17.98)
No	228 (82.02)

Additionally, before providing a culture, a minority of 50 (17.98%) patients had been treated with antibiotics, whereas the remaining 228 (82.02%) patients had not received any antibiotic therapy.

Table 5 presented the ultrasound scan results for the kidney, ureter, and bladder (USS-KUB), which identified obstructive uropathy in 77 (27.69%) respondents. Furthermore, pyelonephritis and a high post-residual volume were each observed in 19 (6.83%) respondents. Prostatomegaly was less common, with USS findings indicating its presence in only 14 (5.03%) respondents.

**Table 5:** Findings in the ultrasound of the kidney, ureter, and bladder among the participants

Variable	Number (%)
Pyelonephritis	19 (6.83)
Obstructive uropathy	77 (27.69)
High post-residual volume	19 (6.83)
Prostatomegaly	14 (5.03)
Not done	149 (53.59)

Table 6 portrays the blood investigations among patients with and without risk factors. Patients with risk factors demonstrated a median C-reactive protein (CRP) level of 163.5 mg/dL, markedly higher than the 86 mg/dL observed in patients without risk factors. The median white blood cell (WBC) count also was higher in the risk group (15.51 vs. 12.35). While both groups had similar capillary blood sugar levels on admission and neutrophil percentages, lymphocyte percentages differed.

**Table 6:** Blood investigation trends with or without risk factors

Variable	With risk factors	Without risk factors
Capillary blood sugar on admission	Median: 120, IQR: 77	Median: 120.5, IQR: 69
Lymphocytes (%)	Median: 9.35, IQR: 14.65	Median: 14.25, IQR: 13
Neutrophils (%)	Median: 79.25, IQR: 18.03	Median: 78.15, IQR: 19.58
White blood cells	Median: 15.51, IQR: 13.47	Median: 12.35, IQR: 6.56
C-reactive protein	Median: 163.5, IQR: 171.75	Median: 86, IQR: 178.75
Hemoglobin (g/dL)	Median: 10.8, IQR: 3.35	Median: 10.45, IQR: 2.48
Platelets	Median: 261.5, IQR: 127.5	Median: 277, IQR: 123.5

Table 7 is regarding the urine full report (UFR) results of the studied population. The number of pus cells was normal in around half (54%) of the participants. However, there were 52 (18.7%) patients whose results had moderately field-full results. Moreover, in 213 (76.6%) patients, there were no red cells in UFR results. The protein levels in UFR were also assessed. There were no proteins in most 130 (46.7%) patients. Furthermore, 183 (65.8%) patients showed positive for organisms in the UFR.

**Table 7:** UFR results in the study population UFR - urine full report

Variable	Number (%)
UFR pus cells	
Moderately field-full	52 (18.7%)
Occasional	76 (27.3%)
Nil	150 (54%)
UFR red cells	
Moderately field-full	10 (3.5%)
Occasional	55 (19.9%)
Nil	213 (76.6%)
UFR protein	
Positive	148 (53.3%)
Nil	130 (46.7%)
UFR organism	
Positive	183 (65.8%)
Nil	95 (34.4%)

Table 8 mentions the association between risk factors, demographic factors, and antibiotic usage before the urine culture. Antibiotics taken before cultures had a significant association with having one of the risk factors, with a p-value of 0.0015. Patients with at least one risk factor above were more likely to receive antibiotics before the urine culture. Likewise, males exhibited a higher prevalence of at least a risk factor than females (p-value 0.0029). There was no significant association between marital status and having a risk factor.

**Table 8:** Association between risk factors, demographic factors and antibiotic usage before urine culture CI - confidence interval; OR - odds ratio

Variable	Risk factors		p-value	OR (95 % CI)
	Yes (N=90)	No (N=188)		
Age	63± 17.983	60 ± 21.260	0.754	
Gender				
Female	39 (43.3)	118 (62.8)	0.0229	0.4536 (0.2689 - 0.7505)
Male	51 (56.6)	70 (37.2)		
Status				
Married	74 (82.2)	157 (83.5)	0.8644	0.9132 (0.4756 - 1.751)
Single	16 (17.8)	31 (16.5)		
Antibiotics taken before cultures				
No	64 (71.1)	164 (87.2)	0.0015	2.776 (1.50 - 5.191)
Yes	26 (28.9)	24 (12.7)		

Table 9 mentions the type of organisms in the culture. *Escherichia coli* was present in most of the sample, 100 (36%). Also, 76 (26%) had *Klebsiella pneumoniae (K. pneumoniae)* subspecies in the culture. Only 39 (14%) of the sample had Gram-negative enteric organisms in their culture. Furthermore, Proteus species were the least found in only five (1.8%) of the culture sample. 

**Table 9:** Prevalence of different organisms in the urine culture

Organisms	Number (%)
Escherichia coli	100 (36%)
*Klebsiella pneumoniae* subspecies	72 (26%)
Gram-negative enteric organism	39 (14%)
Enterococcus species	36 (13%)
Pseudomonas aeruginosa	14 (5%)
Staphylococcus aureus	5 (1.8%)
Proteus species	5 (1.8%)
Other	7 (2.5%)

## Discussion

The mean age of the respondents was 60.0 ± 20.28 years. However, the highest peak was observed at 70 years. The gender distribution in the study is mainly female preponderance. Contrarily, Silver et al. reported that the majority presented were males, but women were more likely to have had a positive urine culture than men [18]. Also, similar results were reported in a cohort study conducted in Spain, where most patients were elderly and female [19]. Also, a higher prevalence of UTIs in females may be due to the short urethra, which could easily cause ascending infections in females.

According to our findings, the majority of respondents didn't have any comorbidities. However, hypertension and diabetes mellitus were seen to be present in most of the respondents with comorbidities. Moreover, a study conducted by Johnson et al. It also revealed that the majority of respondents didn't have any comorbidities [20]. However, Silver et al. reported that the majority of patients (82%) had at least one underlying comorbidity; coronary artery disease (40%) and diabetes mellitus (25%) were among the highest [18]. 

In our study, 62 (22.30%) respondents presented with a fever, and 62 (22.30%) patients presented with lower abdominal pain. Similar findings were observed in a study conducted in India with fever and increased frequency followed by abdominal pain [21]. However, the study conducted by Silver et al. revealed that the patients in their research mainly presented with fever and confusion, which initiated the urine culture [18]. Kumar et al. reported that fever (96.6%) followed by dysuria (20.1%) were the most common symptoms presented for UTI in their study [22]. Furthermore, UTI with symptoms was found to have a positive urine culture more frequently than asymptomatic patients. Especially patients with a fever of more than 38°C or patients with at least one of the following symptoms: dysuria, urgency, frequency, supra-pubic pain, or tenderness [23,24].

In our study, the highest risk factors contributing to the development of UTI were obstructive neuropathy (30 respondents; 10.79%), renal calculi (26 respondents; 9.35%), and indwelling catheters (22 respondents; 7.91%). However, the majority (188 respondents; 67.63%) had no associated risk factors. Chenoweth et al. demonstrated prolonged catheterization, female gender, older age, and diabetes had a very significant association [25]. Moreover, Trautner and Grigoryan pointed out that older age, female sex, and genitourinary tract abnormalities were the main contributors to UTI [26]. Salvatore et al. also had a similar point of view, in which congenital anatomic abnormalities, urinary tract calculi, neurological disorders, diabetes, and indwelling or recurrent bladder catheterizations could lead to UTI [27,28]. 

Our study demonstrated high CRP and leukocytosis among the patients, particularly those with at least a risk factor. A reviewed literature reported a systemic inflammatory response in upper urinary tract infection is present with elevated C-reactive protein, high erythrocyte sedimentation rate, and leukocytosis [29,30]. Moreover, few other studies show that neutrophilia, together with UTI, represents a significantly severe disease following lethal outcomes [31]. In children, leukocytosis with neutrophilia indicates the presence of a UTI caused by *E. coli* commonly [32].

In our study, *Escherichia coli* was present in most of the sample, accounting for 100 participants (36%), followed by the *Klebsiella pneumoniae* subspecies in 72 patients (26%). *Pseudomonas aeruginosa* accounted for only 14 (5%) of cases. However, Proteus species were the least found in the culture sample. Similar results of the majority of culture being *E. coli* were found in several other studies. Also, *E. coli* is the joint causative agent of UTI in women and children [33,34]. Also, Gupta et al. reported that *Klebsiella *and *Pseudomonas *were the most common causative agents after *E. coli *in children [22,35,36]. However, Smith et al. reported that the most common organism found in patients with chronic indwelling catheters was *Enterococcus *spp., and *Pseudomonas aeruginosa* was the second [37]. 

Though our study demonstrated a significant association between males and having at least a risk factor, this finding must be interpreted carefully. Though the female gender per se increases the risk of UTI, as per our study, males should have at least a risk factor to cause a UTI.

It is unclear why patients with at least one risk factor are associated with increased antibiotic consumption before the urine culture. This is likely due to the increased availability of most oral prescription medicines in commercial pharmacies in Sri Lanka.

Limitations

This study encountered several limitations. The data was collected from only one hospital in a specific geographical region, limiting the results' generalizability. Additionally, its hospital-based nature may not reflect practices in outpatient populations. The peak of the COVID-19 pandemic presented a significant challenge, hindering the achievement of the calculated initially full sample size. To validate and expand upon these findings, future research should focus on multi-center, large-sample studies across various regions of Sri Lanka.

## Conclusions

UTIs are a significant health concern, particularly among the elderly, where they pose considerable morbidity. Our findings highlight a heightened susceptibility in women, which could be attributed to inherent anatomical variances such as the shorter urethra in females. Fever and abdominal pain emerged as common symptoms among the study participants, indicating the symptomatic burden of UTIs.

Intriguingly, our data suggest that males who had consumed antibiotics before culture tests were more likely to exhibit risk factors associated with UTIs, pointing toward the complexity of risk profiles across genders. Among the causative agents, *Escherichia coli* and *Klebsiella pneumoniae* were the most frequently identified, underscoring their significant role in UTI pathogenesis. This insight contributes to our understanding of UTI dynamics. It underscores the critical need for targeted strategies in diagnosis, treatment, and, perhaps most importantly, the stewardship of antibiotics to mitigate the risk and impact of UTIs across vulnerable demographics.
